# 

**Published:** 1921-09-03

**Authors:** 


September 3, 1921] [Price Twopence

### The Hospital

The Workers' Newspaper of Administrative Medicine and Institutional Life, Administration, National Insurance and Health.

CONTENTS

**Lord Onslow and Local Committees** ... ... 381

**Insanity and Research...** ... ... ... ... ... 382

**Dangerous Drugs Act, 1920** ... ... ... ... 382

Provisions Relating to Hospitals and Institutions.

**Notes of the Week** ... ... ... ... ... .. 383

The Spählinger Treatment of Tuberculosis—Hospital Week and Pageant: Ideas from America—American Hospital Progress—Power to Contribute to Hospitals—The Costers' Holiday—A Hint to Bolton—The Sale of Property—The Johannesburg Hospital Inquiry—An M.P.'s Conversion—The Patients' Diet at Northampton—Child Critics in Fever Hospitals.

**Diabetes** ... ... ... ... ... ... ... ... 385

A Note on its Treatment and Prevalence.

**The Cure and Control of Cancer** ... ... ... 386

**The Public Well-Being** ... ... ... ... ... ... 387

A Year's Work of the Ministry of Health.

**The Editor's Letter-Box** ... .. ... ... 388

Diplomas in Radiography—"Higher Charges for Distant Patients?"

**The Voluntary Hospitals Commission** ... ... 388

**The Matrons' and Sisters' Department** ... 389

The Nursing Department in the Annual Report.

**Round the Hospitals** ... ... ... ... ... 389

**Practical Points** ... ... ... ... ... ... ... 391

**Institutional Needs** ... ... ... ... ... 391

**Medical History of the War** ... ... ... ... 391

**Welfare Schemes for Industrial Firms** ... ... 391

**The College of Nursing (Limited by Guarantee)** 392

**"Our Day"** ... ... ... ... ... ... ... 392

**Matrons' and Sisters' Appointments** ... ... 392

